# Pakistani Ankylosing Spondylitis Cohort with modifiable cardiovascular risk factors (PAS-CVD) study

**DOI:** 10.12669/pjms.40.3.7265

**Published:** 2024

**Authors:** Abrar Ahmed Wagan, Paras Surahyo

**Affiliations:** 1 Abrar Ahmed Wagan, MBBS, FCPS, FACR Associate Professor of Rheumatology, Indus Medical College, Tando Mohammad Khan, Pakistan; 2 Paras Surahyo, MBBS, FCPS Assistant Professor of Radiology Bilawal Medical College, Jamshoro, Pakistan

**Keywords:** AS, modifiable cardiac risk factors, obesity, high cholesterol, Framingham risk score

## Abstract

**Objective:**

To determine the frequency of modifiable cardiovascular risk factors in the Pakistani cohort with Ankylosing Spondylitis (AS)

**Method:**

After IRB approval, a cross-sectional study was conducted among patients of AS, at the Department of Rheumatology Indus Medical College, Tando Mohammad Khan, from 15^th^ March to 15^th^ September, 2022. After obtaining demographic data, other parameters such as blood pressure (BP) and body mass index were recorded. In addition, a 5 ml blood sample was collected to assess their serum lipid profile, and fasting blood sugar levels. Using the laboratory data, the Framingham cardiovascular risk score was calculated for each patient and they were categorized into low, intermediate, or high-risk categories.

**Results:**

Total 131 cases of ankylosing spondylitis: frequency of modifiable risk factors were: obesity (75.6%), high TG level (62.6%), high risk FRS score (40.5%), high LDL level (38.1%), low HDL (34.4%), hypertension (30.5%), diabetes mellitus (26.7%), high cholesterol level (17.6%), smoking (16%). In univariate analysis AS cases shows that increasing disease duration was associated with more risk of modifiable risk factors (p<0.05), on multivariate analysis, a positive association of age, diastolic blood pressure, smoking, diabetes mellitus, DMARDS, herbal medication-but not statistically significant (p>0.05).

**Conclusion:**

In chronic AS there’s higher prevalence of modifiable cardiovascular risk factors, earlier recognition and effective management helps in prevention of future cardiovascular events.

## INTRODUCTION

Ankylosing spondylitis (AS) is the most common among spondyloarthropathies: psoriatic arthritis, reactive arthritis, spondyloarthritis related to inflammatory bowel disease, and undifferentiated spondyloarthritis.^1^,^2^ AS affects the axial skeleton, presenting as inflammatory low back pain, stiffness, peripheral arthritis, anterior uveitis, enthesitis, affects functionality and quality of life.^3^,^4^ Mortality rate in AS is 1.6-1.9 times higher than the general population, and in 20-40% of cases the cause is either circulatory or cardiovascular diseases.^5^ A Canadian case control study with large population showed, vascular mortality was more in AS than the general population, with 36% higher risk for vascular mortality, when adjusted for baseline cardiovascular risk factors, (hazard ratio (HR) 1.36, 95% confidence interval (CI) (1.13–1.65) and risk stratification was significant among male subjects (HR 1.46, 95%CI 1.13–1.87),compared to controls, patients with AS had a 60% higher risk of cerebrovascular mortality (HR 1.60, 95%CI 1.17–2.20) and a 35% higher risk for cardiovascular mortality (HR 1.35,95%CI 1.07–1.70).^6^

In AS standardized mortality rate (SMR) was significantly elevated for hypertensive and diabetes mellitus (DM) but still unclear: whether it is due to result of cardiac manifestations of disease or a concomitant increase in CV diseases.^7^ There are ongoing vascular, morphologic and functional abnormalities with inflammation-associated endothelial injury, and persistent inflammatory process promotes and leads to atherosclerosis.^8^

In AS there is continuous state of inflammation and there is elevation of pro-inflammatory markers like,IL-6 and CRP and IL-6 itself, induces the acute phase response, leading to elevated CRP, fibrinogen, with activation of monocytes, these deposit fibrinogen in the blood vessel wall and initiates atheroma formation, and in damaged the endothelium is, IL-6 and other chemokine’s are secreted from foam and smooth cells as well, causing even more blood vessel injury, this cytokine also interacts with the hypothalamus-pituitary-adrenal (HPA) axis, influencing cardiac risk factors like insulin sensitivity, elevated BMI, and elevated blood pressure.^3^,^9^ AS not only produce inflammation and atherosclerosis resulting in morbidity and mortality but the risk of multiple comorbidities like hypertension, Type-2 diabetes mellitus and higher prevalence of metabolic syndrome (33%).^7^,^10^ A recent study reported an age-adjusted and sex-adjusted mortality hazard ratio (HR) of 1.60 (95% CI = 1.44-1.77), with increased mortality for men age-adjusted HR = 1.53 (1.36-1.72) and women’s age-adjusted HR = 1.83 (1.50-2.22) in AS and cardiovascular events accounted for (34.7%) of all deaths.^11^

It is clearly evident from research results that cardiovascular events are major cause death in in AS, this study will help in guiding the treating rheumatologists to screen and reduce the risk of traditional cardiac risk factors with establishment of robust strategies for such cases.

## METHODS

This study was conducted at the Department of Rheumatology, Indus Medical College Tando Mohammad Khan. Ankylosing spondylitis diagnosed on (Assessment of spondyloarthritis international society criteria) was selected, prolapse intervertebral disc problems, history of physical trauma, spinal tuberculosis, metastatic spine diseases, and patients using lipid-lowering medications were excluded from this study.

After completing the demographic details, patients were asked in detail about the disease duration, the number of years since the diagnosis, smoking habits, history of herbal medications, body mass index (BMI) was calculated, blood pressure was measured by a trained nurse with a mercury sphygmomanometer in resting position after five minutes mandatory rest, afterward five milliliter of blood was taken by a trained phlebotomist, and blood samples were sent to the laboratory for FBS, Lipid profile analysis on (COBAS-III) Machine. Framingham cardiovascular risk was calculated for each individual, and scores were categorized into low:1-10, intermediate:11-20, high risk>20

### Ethical Approval:

Institutional review board of Indus Medical College Tando Mohammad Khan. Approved the study (IRB/31/2022).

### Statistical Analysis:

Data were stored and analyzed using IBM-SPSS version 23.0. A comparison of mean was done between two groups of spondyloarthropathy duration under and above 8 years, using an independent sample t-test, whereas the Pearson Chi-Square test was used to check the association of qualitative parameters with the duration of disease. Binary logistic regression analysis was done to estimate the odds and 95% confidence interval of risk factors with disease duration, p-values less than 0.05 were considered statistically significant.

## RESULTS

One hundred thirty one cases were divided into two groups. Those with disease duration (> eight years) had mean age 41.1years (SD=±11.1), BMI 28.3Kg/m (SD=±4.8), systolic blood pressure 127.4-mm Hg (SD=±11.1), diastolic blood pressure 84.3mmHg (SD=±9.2), cholesterol 189mg/dl (SD=±45.7). Those with disease duration of < 8 years had mean age 36.1 years (SD=±8.5), BMI 28.0 (SD=±4.3), systolic blood pressure 123.2-mm/Hg (SD=±10.1), diastolic blood pressure 79.6-mmHg (SD=±7.2), cholesterol 181.8 (SD=±34.5), TG 181.7 (SD=±87.2). Other details are given in Table-I. Independent sample t-test showed a significant mean difference between the two groups for Age, SBP, and DBP with p<0.05, (Table-I).

**Table-I:** Baseline Characteristics of Studied Samples (n=131).

Characteristics	Duration ≤8 years (n=75)		Duration >8 years (n=56)		p-value

	Mean	SD	Mean	SD	
Age (years)	36.1	8.5	41.1	11.1	<0.01*
BMI (kg/m2)	28.0	4.3	28.3	4.8	0.74
SBP	123.2	10.1	127.4	11.9	0.03*
DBP	79.6	7.2	84.3	9.2	<0.01*
Cholesterol	181.8	34.5	189.0	45.7	0.30
TG	181.7	87.2	211.4	84.7	0.053
HDL	45.6	6.5	45.7	6.7	0.89
LDL (F)	97.4	28.5	99.7	48.4	0.72
FRS	11.4	11.5	14.2	11.1	0.16

* p<0.05 was considered statistically significant using independent sample t-test.

AS cases with disease duration of >8-years were suffering from hypertension (48.2%) Those on medication included 30.4%) while history of smoking had (17.9%), diabetes mellitus-(33.9%) and those who were using DMARD/NSAID accounted for 17.9%). Similarly patients with disease duration of <8-years had hypertension (17.3%) & 5.3% were on-medication. It also included smokers 14.7% and diabetes mellitus accounted for 21.3%). Among these factors a significant association of hypertension with medication use, TG, LDL, and FRS was observed with the duration of disease, p<0.05. Other details are given in Table-II.

**Table-II:** Association of studied factors with duration of Ankylosing spondylitis.

Characteristics		Duration ≤ 8-years		Duration >8-years		p-value

		n	%	n	%	
Sex	Male	72	96.0	50	89.3	0.13
	Female	3	4.0	6	10.7	
Hypertension with Medication	Yes	4	5.3	17	30.4	<0.01*
Hypertension	Yes	13	17.3	27	48.2	<0.01*
Smoking	Yes	11	14.7	10	17.9	0.62
Diabetes Mellitus	Yes	16	21.3	19	33.9	0.10
DMARDS / NSAID	Yes	47	62.7	37	66.1	0.68
Herbal medication use	Yes	47	62.7	38	67.9	0.53
Family history of CHD	Yes	13	17.3	13	23.2	0.40
BMI Levels	Normal weight	20	26.7	12	21.4	0.60
	Overweight	29	38.7	20	35.7	
	Obese	26	34.7	24	42.9	
Cholesterol	Desirable	63	84.0	45	80.4	0.66
	Borderline High	5	6.7	3	5.4	
	High	7	9.3	8	14.3	
HDL	Low	25	33.3	17	30.4	0.87
	Normal	48	64.0	38	67.9	
	High	2	2.7	1	1.8	
TG	Normal	36	48.0	13	23.2	0.01*
	Borderline high	18	24.0	15	26.8	
	High	20	26.7	28	50.0	
	Very High	1	1.3	0	0.0	
LDL	Optimal	47	62.7	34	60.7	0.01*
	Above Optimal	19	25.3	12	21.4	
	Borderline high	8	10.7	2	3.6	
	High	0	0.0	7	12.5	
	Very High	1	1.3	1	1.8	
FRS	Normal	51	68.0	27	48.2	<0.01*
	Intermediate risk	3	4.0	11	19.6	
	High risk	21	28.0	18	32.1	

* p<0.05 was considered statistically significant using Pearson Chi Square test.

**Fig.1:**
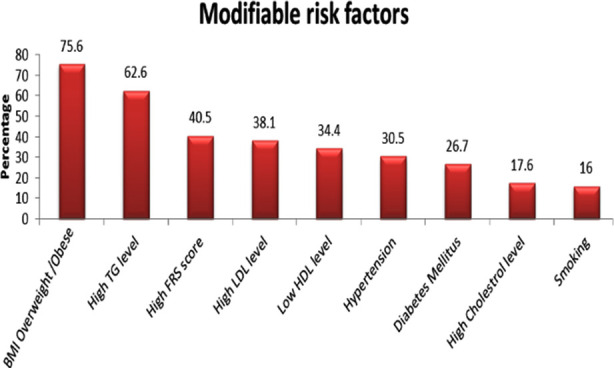
Prevalence of modifiable cardiovascular risk factors.

In univariate analysis AS cases shows that increasing disease duration was associated with more risk of modifiable risk factors (p<0.05). and on multivariate analysis, a positive association of age, diastolic blood pressure, TG, HDL, LDL, diabetes mellitus, FRS, DMARDS, herbal medication-but not statistically significant (p>0.05), (Table-III). Frequency of modifiable risk factors are shown in Fig.1

**Table-III:** Odds ratio with 95% confidence interval using Binary Logistic Regression.

Risk Factors	Univariate Odds Ratio (95% C.I)	Multivariate Odds Ratio (95% C.I)
Age (years)	1.0(1.0-1.0)	1.0(0.9-1.0)
Male	0.3(0.0-1.4)	0.2(0.0-1.1)
BMI (kg/m2)	1.0(0.9-1.0)	0.9(0.8-1.0)
SBP	1.0* (1.0-1.07)	0.9(0.9-1.0)
DBP	1.0*(1.0-1.1)	1.0(0.9-1.1)
Hypertension with medication	7.7*(2.4-24.)	1.9(0.3-9.9)
Hypertension	4.4*(2.0-9.8)	2.5(0.5-11.)
Cholesterol	1.0(0.9-1.0)	0.9(0.9-1.0)
TG	1.0(0.9-1.0)	1.0(0.9-1.0)
HDL	1.0(0.9-1.0)	1.0(0.9-1.1)
LDL	1.0(0.9-1.0)	1.0(0.9-1.0)
Smoker	1.2(0.4-3.2)	0.7(0.2-2.4)
Diabetes Mellitus	1.8(0.8-4.1)	1.1(0.4-3.0)
FRS	1.0(0.9-1.0)	1.0(0.9-1.0)
DMARDS	1.1(0.5-2.3)	1.1(0.4-2.7)
Hakeem / Homeo mediation	1.2(0.6-2.6)	1.0(0.4-2.4)
Family History of CHD	1.4(0.6-3.4)	0.7(0.2-2.3)

Dependent variable (SpA) duration>8-years,

* odds ratio considered statistically significant with p<0.05.

## DISCUSSION

In Ankylosing spondylitis cardiovascular events are the leading cause of mortality ranging from 30 to 50% of all-cause deaths.^12^ A prospective comparative study of AS (radiographic and non-radiographic groups) and general population found an increased risk of ischemic heart disease and stroke compared than the general population.^13^ There are five modifiable cardiovascular risk factors (hypertension, smoking, dyslipidemia, diabetes, and obesity), which account for more than 50% cardiovascular events.^14^

### Arterial Hypertension (AHT):

In various AS studies hypertension was found to be the most frequent treatable cardiovascular risk factor, with a prevalence ranges from (31to 41%).^15^,^16^ In current study hypertension prevalence with disease duration of <8 years was (17.3%) and (5%) were using medications, those with >8 years (48.2%) and (30.4%).

Hypertension in AS could be multifactorial: first, high disease activity, secondly due to decreased mobility because of pain, stiffness, & disability, leading to increased sedentary lifestyle and non-steroidal pain medications.^12^

### Smoking

In AS, worldwide its reported prevalence range is (30 to 40%).^17^ Our study shows the prevalence of smoking with disease duration of <8 years (14.7%) and >8 years (17.9%) much lesser than other reported results.

### Lipid disorder:

Studies have reported increased total and LDL cholesterol with decrease in HDL cholesterol in patients with ankylosing spondylitis.^12^,^18^,^19^

### Diabetes Mellitus:

Several studies have suggested an association between diabetes and peripheral and psoriatic spondyloarthropathy with an increased risk of 1.4 (95% CI = 1.3-1.5) for diabetes.^20^,^21^ In our AS patient’s diabetes prevalence in disease duration of < 8 years (21.3%) and >8 years (33.9%).

### Obesity:

Higher BMI>30/Kg/cm^2^ is not frequently seen in pure axial forms of the disease.^22^ However, the prevalence of obesity is increased in patients with peripheral forms of spondyloarthropathy and has been reported to involve 30% of patients with psoriatic spondylitis. In this study cohort, overweight to obesity was present in (38.7%) cases with disease duration of <8 and 35.7% in duration >8 years and obesity being the highest prevalent modifiable risk factor (75%) and the reason could be the increased and illicit use of herbal medications in these cases.

### Framingham cardiovascular risk:

In our cohort high framingham cardiovascular risk score; in cases with disease duration of < 8 years high was (28%) and in >8 years was (32.1%). The incidence of myocardial infarction in AS patients was 7.4%, while the incidence in the control group was 4.6%, but this did not achieve a level of significance in the meta-analysis.^23^,^24^ Cardiovascular diseases are thought to be more prevalent in patients with AS due to the systemic inflammatory nature, decreased levels of HDL cholesterol, increased prevalence of metabolic syndrome and the long-term use of NSAIDs may contribute to it.^25^,^26^ Pakistani studies have reported the frequency of AS around (1%), which was higher than those reported in earlier COPCORD studies (0.07 to 0.26%),as per geographical prevalence (0.1/1000) in northern and (0.9/1000) in southern parts of Pakistan.^27^,^28^

### Strengths and Limitations:

This is a single center study, with small sample size so, the results can’t represent the entire population; its strength is the first study from Pakistan, which estimated the ten years Framingham cardiovascular risk score with prevalence of modifiable cardiovascular risk factors in AS.

## CONCLUSION

In AS patients, regular follow-up, screening for dyslipidemia, cardiovascular risk assessment with effective management, and counseling for adaptation of healthy life style, may reduce the risk of future cardiovascular events.
